# Structure of the Sea-serpent

**Published:** 1847-06

**Authors:** 


*Structure of the Sea Serpent.*—At the last converzatione of the Warren Club, an association of literary and scientific gentlemen of this city, Prof. Agassiz gave his views of the probable external structure of the far-famed sea serpent, whose visits on our coast are placed beyond doubt by a chain of concurrent testimony strong enough to establish any fact. It is the opinion of this eminent naturalist, that the extraordinary reptile so often described by seafaring people and others, is intermediate in structure and organization between the ichthyosaurus and plesiosaurus, monsters that lived at an extremely remote and undefined geological period in the history of our earth. He supposes that this marine nondescript must have paddles, like those ancient fish lizards, but is uncertain in regard to the mode of respiration, whether it is effected by lungs or gills. That point has not yet been determined in his mind in regard to the plesiosaurus, and he has lately written to Mr. Owen on the subject. It was the unqualified opinion of Cuvier, however, that the plesiosaurus not only breathed air, but that it also had very capacious lungs. From the circumstance that the ribs bear a striking resemblance to those of the chamelion, the great French naturalist suggested that the animal might have been a kind of marine chamelion, having the power of changing its color, and thus eluding the pursuit of its rapacious and formidable enemies. Such a property would be in accordance with the general law of compensation,—its jaws being both small and weak, and it had neither claws nor a long tail for defence; but the great serpent is supposed to have both the first and last of those organs to perfection. Whenever the sea serpent is taken, a feat which we trust will ultimately be accomplished, it would be curious if an anatomical examination should prove that Prof. Agassiz actually predicted the true plan of its construction. This same gentleman re-constructed, on paper, a fish of the primeval world, without having any other part of the animal than a single, solitary scale. When, at length, the fish was found, his drawing corresponded precisely with nature's own work. Such is the accuracy of modern science.—*Boston Med. and Surg. Journal.*

